# Screening high-risk population of persistent postpartum hypertension in women with preeclampsia using latent class cluster analysis

**DOI:** 10.1186/s12884-022-05003-4

**Published:** 2022-09-06

**Authors:** Yuan-Yuan Li, Jing Cao, Jia-Lei Li, Jun-Yan Zhu, Yong-Mei Li, De-Ping Wang, Hong Liu, Hai-Lan Yang, Yin-Fang He, Li-Yan Hu, Rui Zhao, Chu Zheng, Yan-Bo Zhang, Ji-Min Cao

**Affiliations:** 1 Key Laboratory of Cellular Physiology at Shanxi Medical University, Ministry of Education, Taiyuan, China; 2 Department of Physiology, Shanxi Medical University, Taiyuan, China; 3 Department of Critical Care Medicine, The First Hospital of Shanxi Medical University, Taiyuan, China; 4 Department of Maternity, The First Hospital of Shanxi Medical University, Taiyuan, China; 5 Department of Obstetrics Gynecology, Shanxi Children’s Hospital and Women Health Center, Taiyuan, China; 6 Department of Clinical Laboratory, Shanxi Children’s Hospital and Women Health Center, Taiyuan, China; 7 Division of Health Statistics, School of Public Health, Shanxi Medical University, Taiyuan, China

**Keywords:** preeclampsia, postpartum hypertension, cardiovascular disease, latent class cluster analysis

## Abstract

**Background:**

A significant proportion of women with preeclampsia (PE) exhibit persistent postpartum hypertension (PHTN) at 3 months postpartum associated with cardiovascular morbidity. This study aimed to screen patients with PE to identify the high-risk population with persistent PHTN.

**Methods:**

This retrospective cohort study enrolled 1,000 PE patients with complete parturient and postpartum blood pressure (BP) profiles at 3 months postpartum. The enrolled patients exhibited new-onset hypertension after 20 weeks of pregnancy, while those with PE superimposed upon chronic hypertension were excluded. Latent class cluster analysis (LCCA), a method of unsupervised learning in machine learning, was performed to ascertain maternal exposure clusters from eight variables and 35 subordinate risk factors. Logistic regression was applied to calculate odds ratios (OR) indicating the association between clusters and PHTN.

**Results:**

The 1,000 participants were classified into three exposure clusters (subpopulations with similar characteristics) according to persistent PHTN development: high-risk cluster (31.2%), medium-risk cluster (36.8%), and low-risk cluster (32.0%). Among the 1,000 PE patients, a total of 134 (13.4%) were diagnosed with persistent PHTN, while the percentages of persistent PHTN were24.68%, 10.05%, and 6.25% in the high-, medium-, and low-risk clusters, respectively. Persistent PHTN in the high-risk cluster was nearly five times higher (OR, 4.915; 95% confidence interval (CI), 2.92–8.27) and three times (OR, 2.931; 95% CI, 1.91–4.49) than in the low- and medium-risk clusters, respectively. Persistent PHTN did not differ between the medium- and low-risk clusters. Subjects in the high-risk cluster were older and showed higher BP, poorer prenatal organ function, more adverse pregnancy events, and greater medication requirement than the other two groups.

**Conclusion:**

Patients with PE can be classified into high-, medium-, and low-risk clusters according to persistent PHTN severity; each cluster has cognizable clinical features. This study’s findings stress the importance of controlling persistent PHTN to prevent future cardiovascular disease.

**Supplementary Information:**

The online version contains supplementary material available at 10.1186/s12884-022-05003-4.

## Introduction

Preeclampsia (PE) is a common and severe complication of pregnancy that manifests as new-onset hypertension and proteinuria and can progress to severe PE with multi-organ involvement [1–3]. An estimated 2-8% of pregnant women worldwide suffer from PE [4, 5]. In the last decade, mounting evidence has suggested that preeclamptic women are susceptible to developing cardiovascular disease (CVD) later in life [6–8].

Maternal hypertension and proteinuria usually disappear in most PE patients within the first week postpartum; until 3 months, blood pressure (BP) mostly returns to normal [1, 9, 10]. However, about 20% of patients with PE exhibit persistent postpartum hypertension (PHTN), and the occurrence of some forms of PHTN, including sustained hypertension, masked hypertension, and white-coat hypertension, could be as high as 41.5% at 1 year after discharge in patients with severe PE [11–13], who require long-term antihypertensive medication. One proposed risk factor for cardiovascular morbidity is the persistence of hypertension in the postpartum period [14, 15]. Hypertensive women who experience PE are reportedly at a two-fold risk of developing CVD in the next decades compared to those with PE but become normotensive after delivery [16]. Notably, postpartum follow-up of PE is inadequate, with reported rates of 20-60%, and a large proportion of obstetricians neglect to follow up postpartum BP in PE patients [13, 15, 17, 18]. Therefore, early screening for those at high risk of developing persistent PHTN helps clinicians provide accurate postpartum BP monitoring and timely intervention for in such patients.

PE is associated with multiple risk factors [2], and its risk assessments require methods that can integrate them. Current PE models mostly involve the early prediction of adverse pregnancy outcomes and long-term cardiovascular disease risk [19–21]. Prognostic data on BP profiles at short-term follow-up during the postpartum period in patients with PE are scarce, although some other risk factors of PHTN have been suggested, including preexisting hypertension before pregnancy and a higher body mass index (BMI) [11], older age, smoking, pre-pregnancy obesity, comorbidities such as thyroid disorders [12], and decreased serum placental growth factor [22]. Few studies to date have reported the clustered and combined effects of multiple risk factors on BP recovery during the 3-month or longer period after delivery in patients with PE. It remains unclear whether PE increases the occurrence of PHTN through the known risk factors associated with PE and CVD. The present study aimed to identify the categories of PE patients using latent class cluster analysis (LCCA) by combining multiple risk factors. Unlike traditional single-factor approaches, LCCA is a machine learning method and has certain advantages; for example, it can explore the interrelationships among multiple risk factors and classify similar objects into groups, and thus can be applied for screening high-risk populations [23, 24]. This study may help identify high-risk clusters in the PE population and provide appropriate treatment strategies for those who may develop persistent PHTN and CVD.

## Methods

### Study population

**Fig. 1:**
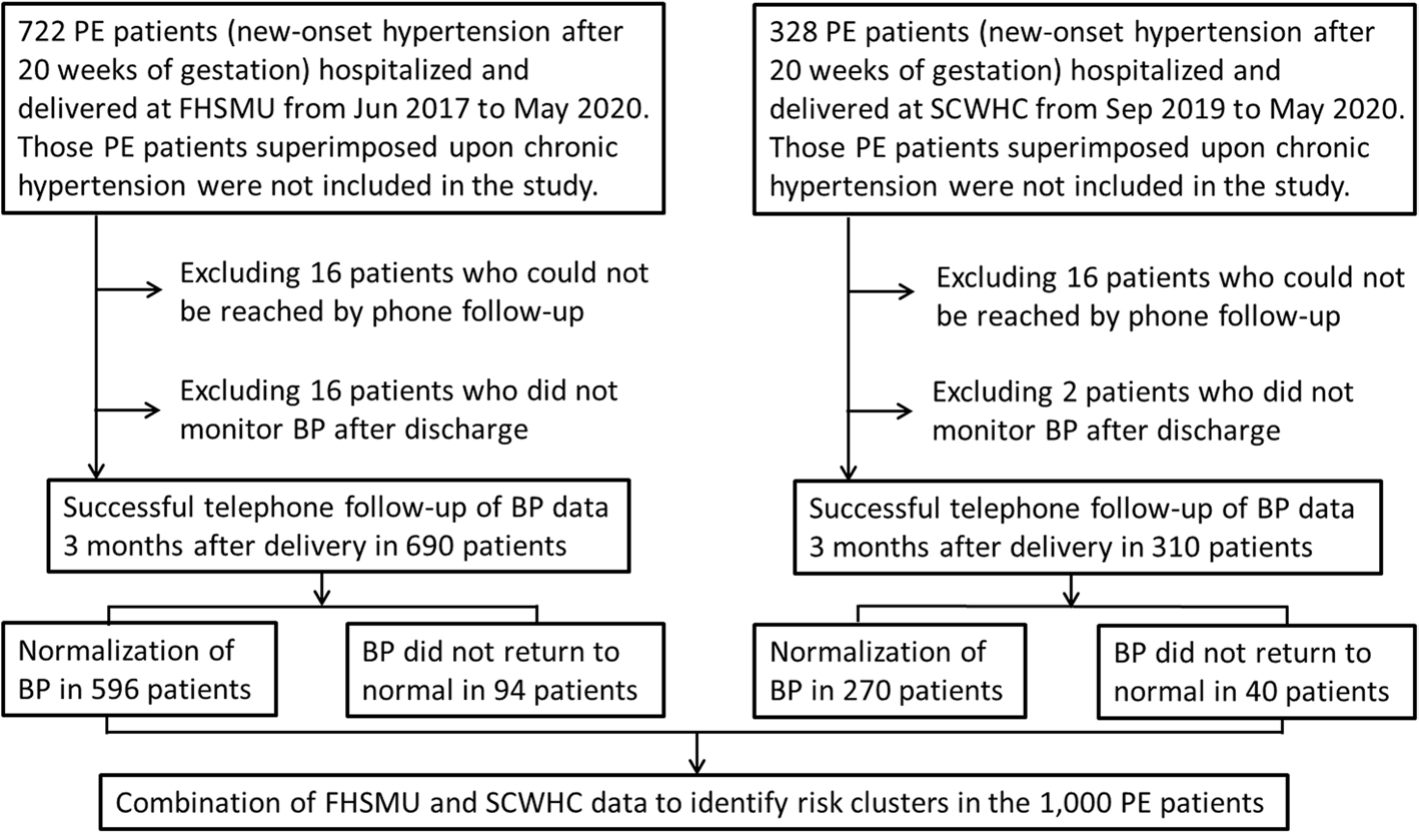
Study flowchart outlining the composition of final PE cohort using the datasets from the First Hospital of Shanxi Medical University (FHSMU) and Shanxi Children’s Hospital and Women Health Center (SCWHC)

This retrospective multicenter cohort study recruited patients from the First Hospital of Shanxi Medical University (FHSMU) between June 2017 and May 2020 and Shanxi Children's Hospital and Women Health Center (SCWHC) from September 2019 to May 2020 with a confirmed diagnosis of PE or who developed PE after admission. A study flowchart is shown in Fig. 1. The diagnosis of PE conformed to the 2018 definition of International Society for the Study of Hypertension in Pregnancy and ACOG 2019 [1, 2]. PE is characterized by new-onset of hypertension (systolic BP ≥ 140 mmHg and/or diastolic BP ≥ 90 mmHg) and exhibits at least one of the following new-onset symptoms during or after 20 weeks of gestation: 1) proteinuria (24-h urinary protein ≥ 300 mg/day or dipstick reading ≥ 2+); 2) other maternal organ dysfunction, such as acute kidney injury (creatinine ≥ 90 μmol/L or 1.0 mg/dL), hepatic dysfunction (alanine aminotransferase or aspartate aminotransferase > 40 IU/L, with or without epigastric abdominal or right upper quadrant pain), neurological dysfunction (such as eclampsia and change in mental status), or hematologic complications (blood platelet count < 150,000/μL, disseminated intravascular coagulation, or hematolysis); and 3) uteroplacental complications (abnormal Doppler waveform of the umbilical artery, fetal growth restriction, or stillbirth). Patients who were diagnosed with PE superimposed upon chronic hypertension, pre-pregnancy hypertension, or hypertension that occurred within the first 20 weeks of pregnancy were not included in the study.

The study flowchart and patient information are presented in Fig. 1. Between June 2017 and May 2020, a total of 722 PE patients who were admitted to the FHSMU and ended their pregnancies were included, excluding 16 without follow-up and 16 without postpartum BP measurement data. Thus, 690 of the 722 PE patients treated at the FHSMU were included; the other 32 were excluded. Of the 690 patients from the FHSMU, the BP data of 94 (13.62%) remained higher at 3 months postpartum. Among patients treated at the SCWHC between September 2019 and May 2020, 328 with PE ended their pregnancies, excluding 16 patients without follow-up and two without postpartum BP measurement data; the remaining 310 were included in the study. Among the 310 patients in the SCWHC group, the BP values did not return to normal by 3 months postpartum in 40 (12.90%). Thus, 1,000 (690 + 310) PE patients from the two hospitals were included, while 50 were excluded due to lack of postpartum BP data or phone interview failure.

### Defining persistent PHTN

The recruited PE patients were followed up by telephone interviews to confirm persistent PHTN at 3 months postpartum. BP was measured twice a day by trained nurses in community clinics and/or by trained family members at home using mercurial or electronic arm cuff BP meters after a 5-min rest. BP measurements were repeated three times within 10 min, with a 1-min interval between measurements. Mean systolic and diastolic BP values were recorded. In the present study, patients with persistent PHTN were defined as those who experienced PE and still showed hypertension (average systolic BP ≥ 140 mmHg; average diastolic BP ≥ 90 mmHg) within 3 months postpartum and the requirement for cardiovascular consults for further investigation and medication. The end date of follow-up was October 2020. Patients with BP that did not return to normal by 3 months postpartum were included as outcome events. Patients who could not be reached by telephone for follow-up and whose BP was not monitored within 3 months postpartum were excluded from the study cohort.

### Data collection

The data and diagnoses of the enrolled patients were collected, including maternal demographic characteristics and relevant clinical laboratory tests performed within 7 days prior to the end of pregnancy. If the index visit involved multiple tests, the worst value was selected. A total of 35 candidate risk factors, including laboratory test results, were entered from the literature reviews [1, 2, 20, 25–27]. Laboratory indicators were converted from continuous to categorical variables based on whether they were outside the normal range (Table 1).

**Table 1:** Description of the eight indicator variables for LCCA and the baseline characteristics of the 1,000 enrolled PE patients

Indicator variable	Risk factors	YES	NO	MIN	MAX	Persistent PHTN (134)	No PHTN (866)	*P*	References
1. Maternal delivery age	Maternal delivery age			16	46	32 (28,36)	30 (27,33)	< 0.001	[1, 2, 20]
2. Mean arterial pressure	Mean arterial pressure (MAP) (mmHg)			91	180	126.67 (116.67,137.33)	117.33 (110,126.67)	< 0.001	[1, 2, 20]
3. Drugs use	Beta blockers	1	0	0	4	59 (44.03)	218 (25.17)	< 0.001	[1, 2, 20]
	Calcium antagonist	1	0			29 (21.64)	89 (10.28)	< 0.001	
	Dexamethasone	1	0			11 (8.21)	42 (4.85)	0.106	
	MgSO_4_	1	0			30 (22.39)	77 (8.89)	< 0.001	
4. Medical history	Body mass index (BMI) before pregnancy ≥ 25	1	0	0	12	63 (47.01)	329 (37.99)	0.046	[1, 2, 20, 25–27]
	Prior preeclampsia	1	0			9 (6.72)	55 (6.35)	0.872	
	History of heart / kidney disease	1	0			2 (1.49)	7 (0.81)	0.435	
	Family history of hypertension	1	0			25 (18.66)	124 (14.32)	0.189	
	Number of abortions	1-3	0					0.201	
	0					64 (47.76)	489 (56.47)		
	1					49 (36.57)	248 (28.64)		
	2					13 (9.70)	90 (10.39)		
	≥ 3					8 (5.97)	39 (4.5)		
	Number of births	1-3	0					0.177	
	0					68 (50.75)	523 (60.39)		
	1					54 (40.30)	286 (33.03)		
	2					11 (8.21)	49 (5.66)		
	≥ 3					1 (0.75)	8 (0.92)		
	≥ 10 years from the previous birth	1	0			30 (22.39)	94 (10.85)	< 0.001	
	PE was diagnosed before 32 weeks of gestation	1	0			61 (45.52)	295 (34.06)	0.010	
5. Adverse pregnancy outcome	Preterm birth	1	0	0	6	90 (67.16)	459 (53)	0.002	[1, 2, 20]
	Postpartum hemorrhage	1	0			7 (5.22)	42 (4.85)	0.852	
	Pericardial or pleural effusion	1	0			15 (11.19)	23 (2.66)	< 0.001	
	Placental abruption	1	0			18 (13.43)	94 (10.85)	0.378	
	Low birth weight infants / fetal growth restriction	1	0			24 (17.91)	130 (15.01)	0.387	
	HELLP syndrome	1	0			20 (14.93)	50 (5.77)	< 0.001	
6. Blood cell and coagulation test	Platelet count (< 100×10^9^/L)	1	0	0	5	12 (8.96)	53 (6.12)	0.215	[1, 2, 20, 27]
	Neutrophil count (> 6.3×10^9^/L)	1	0			83 (61.94)	472 (54.50)	0.107	
	Monocyte count (> 0.6×10^9^/L or < 0.1×10^9^/L)	1	0			51 (38.06)	283 (32.68)	0.219	
	PT% (> 130% or < 70%)	1	0			57 (42.54)	311 (35.91)	0.139	
	INR (>1.15 or < 0.85)	1	0			26 (19.40)	150 (17.32)	0.556	
7. Liver and renal function	AST (> 40 U/L)	1	0	0	9	29 (21.64)	128 (14.78)	0.042	[1, 2, 20, 27]
	ALT (> 40 U/L)	1	0			13 (9.70)	69 (7.97)	0.496	
	ALB (< 30 g/L)	1	0			65 (48.51)	346 (39.95)	0.061	
	Proteinuria	1-4	0					0.308	
	0					20 (14.93)	123 (14.22)		
	1+					26 (19.40)	237 (27.40)		
	2+					29 (21.64)	149 (17.23)		
	3+					49 (36.57)	307 (35.49)		
	4+					10 (7.46)	49 (5.66)		
	Serum creatinine (> 1.0 mg/dL)	1	0			11 (8.21)	49 (5.66)	0.247	
	Serum urea nitrogen (> 7.6 mmol/L)	1	0			9 (6.72)	45 (5.20)	0.469	
8. Blood myocardial enzyme and electrolyte test	Serum creatine kinase (> 200 U/L)	1	0	0	4	14 (10.45)	69 (7.97)	0.333	[1, 2, 27]
	Serum lactate dehydrogenase (> 250 U/L)	1	0			47 (35.07)	263 (30.37)	0.273	
	Serum potassium (> 5.5 mmol/L or < 3.5 mmol/L)	1	0			7 (5.22)	35 (4.04)	0.525	
	Serum calcium (< 2.11 mmol/L)	1	0			60 (44.78)	297 (34.30)	0.018	

*MAP* Mean arterial pressure, expressed as median (25% quartile, 75% quartile), i.e., [M (P25, P75)]. *BMI* Body mass index, *HELLP syndrome* Hemolysis, elevated liver enzymes, low platelets syndrome, *PT%* prothrombin activity (%), *INR* International normalized ratio, *AST* Aspartate aminotransferase, *ALT* Alanine aminotransferase, *ALB* Albumin

### Latent class cluster analysis

The 35 candidate risk factors were categorized into eight important indicator variables, including maternal delivery age, mean arterial pressure (MAP = diastolic BP + 1/3 pulse pressure difference; maximum MAP levels during pregnancy were used in the study), drug use during pregnancy, medical history, adverse pregnancy outcomes, blood cell and coagulation tests performed within 7 days before delivery, altered liver and renal functions within 7 days before delivery, and elevated blood myocardial enzymes and electrolyte disbalance within 7 days before delivery. Among the 35 risk factors, no categorical variable data were missing. Some continuous variable data were missing, including up to 2.5% of those for serum creatine kinase and serum lactate dehydrogenase, while data for prothrombin activity (%), international normalized ratio, albumin, serum creatinine, serum urea nitrogen, and serum potassium were missing for fewer than five cases; instead, mean values were used. Each of the eight indicator variables except maternal age and MAP included multiple risk factors. These variables were aggregated and assessed as total risk factor scores, with a dimensionality reduction of 0-N (Table 1) [28]. All eight indicators were considered continuous variables and standardized by the LCCA.

LCCA, a model-based clustering approach, was conducted to analyze the eight indicator variables using R 3.6.1 software. It assumes that heterogeneous populations are a mixture of populations; that is, a latent class is used to classify populations. This method classifies the population by probability; that is, the individual belongs to a cluster with a certain probability, and the individual is ultimately assigned to the cluster with the highest posterior probability [29]. LCCA for categorical indicator variables is called latent class analysis, while that for continuous indicator variables is called latent profile analysis (LPA). The eight indicators we studied were continuous variables, and the basic principle of LPA was to suppose that the probability density function of the P-dimensional continuous manifest variable vector *y*_*i*_ can be expressed as the following equation 1:

1 $$f\left({y}_i\right)=\sum_{k=1}^K{\eta}_k{f}_k\left({y}_i\left|{\mu}_k,{\Sigma}_k\right.\right)$$

Here *η*_*k*_ denotes the latent class probabilities and *K* is the number of clusters (= 1, 2, …, *K*); *y*_*i*_ is the score of object *i* on a set of manifest variables, assuming that within the cluster *k*, *y*_*i*_ came from an independent multivariate normal distribution; *μ*_*k *_is the mean vector; and ∑_*k*_ is the variance-covariance matrix. After model establishment using Bayesian theory, the posterior probability of assigning patients to class *k* was calculated using the following equation 2:

2 $$P\left(k\left|{y}_i\right.\right)=\frac{\eta_k{f}_k\left({y}_i\left|{\mu}_k,{\Sigma}_k\right.\right)}{\sum_{k=1}^K{\eta}_k{f}_k\left({y}_i\left|{\mu}_k,{\Sigma}_k\right.\right)}$$

LPA with the mclust package was used to define clusters of participants with similar clinical profiles. We used mclustBIC to observe the Bayesian Information Criterion (BIC) for different profiles and the integrated completed likelihood (ICL) to penalize the model’s instability to stabilize the number of obtained models. Finally, PE patients were classified into different latent classes.

### Statistical analysis

Continuous variables in the baseline information are expressed as median and quartile [M (P_25_, P_75_)], and comparisons between PE patients with versus without persistent PHTN were made using the Mann-Whitney U test. Categorical variables are expressed as count and percentage, and the chi-squared test was used to compare PE patients with versus without persistent PHTN. Standardized characteristics of clusters are expressed as mean ± standard deviation (SD), while cluster comparisons were performed using analysis of variance. The logistic regression analysis was performed to explore the association between exposure clusters and persistent PHTN. The statistical analysis was performed using SPSS 22.0, and statistical significance was set at *P* < 0.05. Bonferroni correction was used to adjust the *P* values for multiple tests.

## Results

### Baseline characteristics of PE patients

Indicator variables and patients’ baseline characteristics are presented in Table 1. Except for the first and second indicator variables (maternal delivery age and MAP, respectively), all indicator variables consisted of 33 risk factors that were aggregated into a score for each indicator variable according to the presence or absence of the corresponding risk factor. The scoring standards (a series of yes/no questions) are shown in the *Data collection* section of the Methods section.

Among the 1,000 PE subjects, 134 developed persistent PHTN and required transfer to a cardiovascular department for further evaluation and antihypertensive medication. Maternal delivery age, MAP, and prenatal use of antihypertensive drugs in the 134 patients with PHTN were significantly higher than those in the 866 patients without PHTN (*P* < 0.001, Mann-Whitney U test and chi-squared test). Compared with normotensive subjects at 3 months postpartum, patients who developed persistent PHTN showed PE features earlier (earlier than 32 weeks’ gestation), longer intervals between births (/pregnancies) (> 10 years), a higher incidence of adverse pregnancy outcomes, and worse laboratory results.

### LCCA results

Table S1 presents the analytical results of the five models assessed for goodness of fit. The BIC model 2- and model 3-clusters were smaller, while the model 3-cluster was more suitable for screening high-risk study populations.

According to the model 3-cluster, three clusters of maternal exposure were identified and the standardized values of the eight indicator variables were compared (Table 2). The risk factors among the three clusters are shown in Table S2. The numbers (percentages) of 1,000 PE patients distributed in the three clusters are as follows: cluster 1 (low-risk), 320 (32.0%); cluster 2 (medium-risk), 368 (36.8%); and cluster 3 (high-risk), 312 (31.2%).

**Table 2:** Comparison of standardized characteristics among the three clusters of PE patients

Indicator variables	Low-risk cluster (cluster 1, 320)	Medium-risk cluster (cluster 2, 368)	High-risk cluster (cluster 3, 312)	F	*P*	Multiple comparisons
Maternal delivery age	-0.05±0.88	-0.06±1.01	0.13±1.09	3.376	0.024	3 > 2
Mean arterial pressure	-0.63±0.58	-0.06±0.88	0.72±1.01	203.341	< 0.001	3 > 2 > 1
Drugs use	-0.43±0.47	-0.57±0.31	1.12±1.02	622.598	< 0.001	3 > 1 > 2
Medical history	-0.24±0.87	-0.10±0.90	0.36±1.13	33.610	< 0.001	3 > 2, 3 > 1
Outcome ^a^	-0.71±0.56	0.25±1.06	0.43±0.89	160.049	< 0.001	3 > 2 > 1
B and C test ^b^	-0.21±0.87	-0.01±1.04	0.22±1.04	14.452	< 0.001	3 > 2 > 1
Liver and renal function	-0.55±0.80	0.04±0.93	0.52±0.97	110.936	< 0.001	3 > 2 > 1
Mye and Elec test ^c^	-0.90±0.00	0.36±0.95	0.50±0.95	316.834	< 0.001	3 > 2 > 1

^a^ Outcome = Adverse pregnancy outcome. ^b^ B and C test = Blood cell and coagulation test.

^c^ Mye and Elec test = Myocardial enzyme and electrolyte test.

**Fig. 2:**
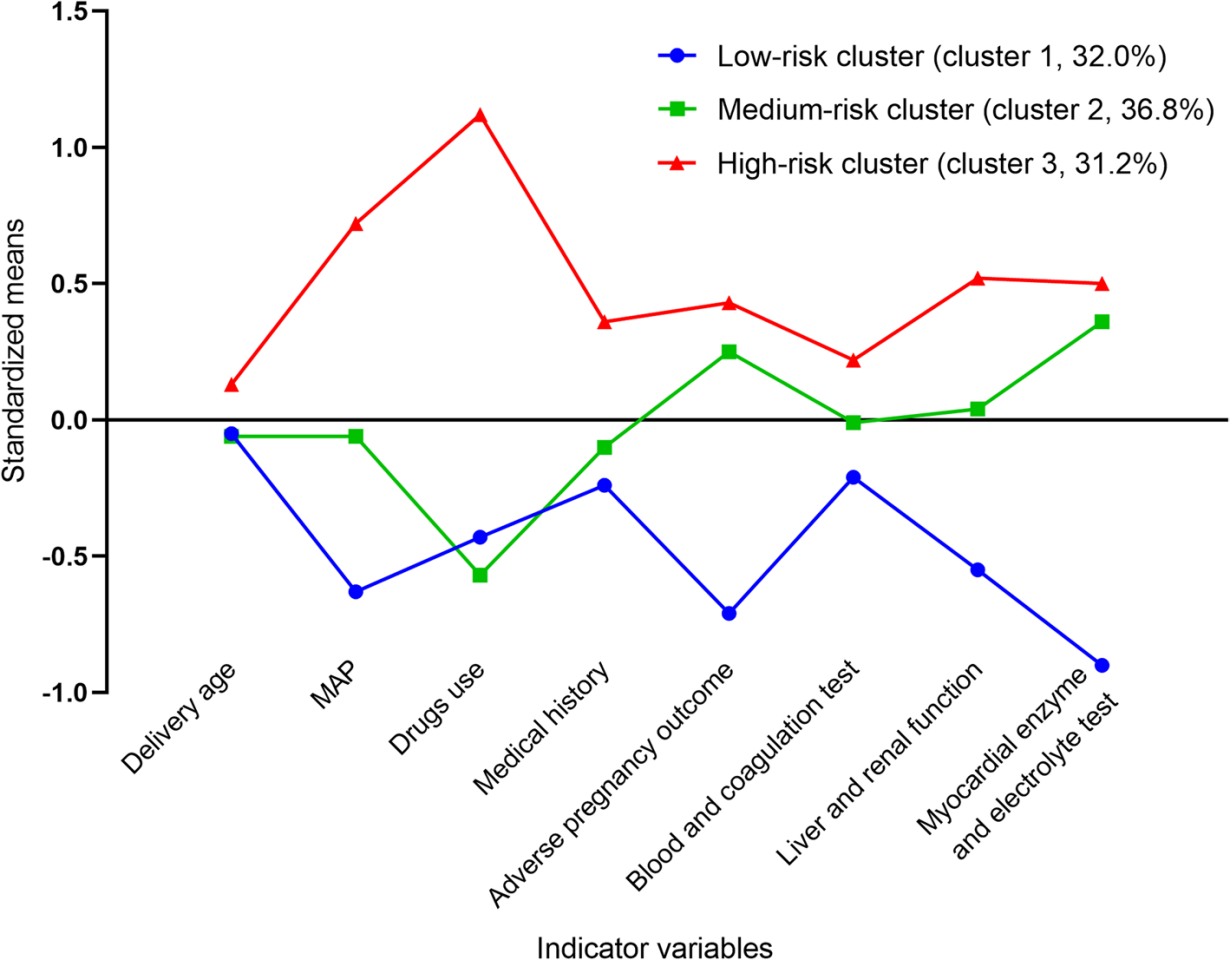
Standardized means of the indicator variables for different risk clusters. Cluster 1 indicated the low-risk cluster, cluster 2 was the medium-risk cluster, and cluster 3 was the high-risk cluster

Cluster 3 exhibited the highest levels of the eight indicator variables. Thus, cluster 3 was characterized as a high-risk cluster. Cluster 2 did not differ from cluster 1 in maternal delivery age and medical history indicators and had a lower drug use indicator level, but higher levels of the remaining five indicators than cluster 1. Consequently, cluster 2 was characterized as a medium-risk cluster, while cluster 1 was a low-risk cluster. Figure 2 shows the normalized mean values of the eight indicator variables in the three clusters. There were statistically significant differences among the eight indicator variables in the high-, medium-, and low-risk clusters.

Each patient with PE was assigned to the most likely cluster based on the parameters constructed by the trichotomous model. Table S3 describes the standardized values and LCCA cluster assignments for selected patients with PE.

### Association of persistent PHTN with identified latent classes

The event rate of persistent PHTN was significantly higher in the high-risk cluster (24.68%) than in the medium-risk (10.05%) and low-risk (6.25%) clusters, and all statistical differences were significant, but the differences in the latter two were statistically insignificant (Table 3). In addition, the probability of persistent PHTN in the high-risk population was 4.915 and 2.931 times higher than that in the low- and medium-risk populations, respectively. The probability of persistent PHTN was 1.677 times higher in the medium- versus low-risk population, but the difference was statistically insignificant (Table 3).

**Table 3:** Event rates of PHTN in different clusters and associations of PHTN with risk clusters

Clusters	N	Event (%)	χ^2^	OR (95% CI)^a^	OR (95% CI)^b^
High-risk cluster (cluster 3)	312	77 (24.68) ^*#^	51.854	4.915 (2.92, 8.27) ^△^	2.931 (1.91, 4.49) ^△^
Medium-risk cluster (cluster 2)	368	37 (10.05)		1.677 (0.95, 2.95)	1 (ref)
Low-risk cluster (cluster 1)	320	20 (6.25)		1 (ref)	
Total	1000	134 (13.4)			

^*^ *P* < 0.001 *vs*. low-risk cluster. ^#^ *P* < 0.001 *vs*. medium-risk cluster. ^△^ *P* < 0.001. ^a^ Referent (ref) was low-risk cluster. ^b^ Referent (ref) was medium-risk cluster.

## Discussion

The present study investigated the trends in BP changes and other cardiovascular risk factors in 1,000 PE patients at 3 months postpartum, mainly using the LCCA method. We found that these PE patients showed differential risks of persistent PHTN and could be subdivided into high-, medium-, and low-risk clusters according to persistent PHTN severity as indicated by the eight indicator variables and the subordinate 35 risk factors shown in Table 1. Overall, approximately 13.40% of patients with PE exhibited persistent PHTN at 3 months postpartum. The incidence of persistent PHTN in the high-risk cluster was nearly five and three times higher than those in the low-risk and medium-risk clusters, respectively. Those who developed persistent PHTN were usually older; had higher BMI and MAP values; used antihypertensive drugs before delivery; and had abnormal pregnancy features (including earlier onset of PE and higher abortion rate), larger birth numbers, longer intervals between births, and worse laboratory results. These findings may help clinicians and patients realize the severity of persistent PHTN, thus strengthening the importance of follow-up, timely intervention, and improved patient awareness of postpartum BP self-regulation. As a result, such monitoring and treatment may reduce the incidence of long-term CVD in women.

PE is a severe hypertensive disorder of pregnancy (HDP). The follow-up of HDP patients for 5-21 years after delivery demonstrated that they had a four-fold higher risk of developing PHTN than those without HDP [6, 8, 21]. One-year follow-up studies showed that PHTN occurred in 17-29% of patients with PE after discharge and that the probability of postpartum CVD was significantly higher in the PE group than in those with normal pregnancies; among those with PE, the BP returned to normal [12, 13, 22, 30]. The 13.4% incidence of PHTN among the 1,000 PE subjects here reflects the morbidity of persistent PHTN in Taiyuan City, Shanxi Province, China.

An appropriate approach may help improve evaluations of PHTN severity and subsequent cardiovascular events in pregnant women after delivery. A combination of maternal and fetal parameters may reportedly detect the higher risk of PE [31]. The evaluation and management of PHTN requires a multidisciplinary approach [10]. Studies of cardiovascular risk factors before versus after pregnancy reported that half of the increased risk of future hypertension in women with PE is attributable to prenatal risk factors [32]. LCCA, an unsupervised machine learning method, can be used to screen high-risk populations. LCCA assumes that a heterogeneous group consists of a mixture of aggregates, and a latent class variable determines the optimal model. With the help of goodness-of-fit evaluation metrics, LCCA may achieve “dimensionality reduction” at the variable level and cluster at the individual level. The latent classes extracted by LCCA both reflect the comprehensive effects of different influencing factors and aid the further analysis of the characteristics of different population clusters. No single current test can reliably predict the risk of PE. As a multidisciplinary approach that integrates multiple risk factors, LCCA has certain advantages over single tests for evaluating the risks of pregnancy-associated diseases in later life. LCCA has been used to identify high-risk populations requiring clinical treatment and identify distinct subgroups within the clinical risk population [33, 34]. LCCA has also been used effectively to screen populations at high risk of birth defects [35, 36]. Using LCCA, we successfully clustered the subpopulations of patients with PE in terms of persistent PHTN and identified the characteristics of each clustered population. This assessment model can be applied to assess the risk of persistent PHTN.

### Strengths and limitations

The present study’s main strengths were as follows: 1) over 1,000 PE patients were screened; and 2) the LCCA assessment model had good accuracy, could be applied to evaluate persistent PHTN in PE patients, and may help establish early and precise guidance for managing persistent PHTN and reducing the risk of future CVD. The study also had some limitations, including: 1) its relatively shorter follow-up time duration (3 months); 2) potential bias caused by its relatively small cohort size; and 3) its retrospective study design inevitably leading to incomplete medical history data, such as routine testing of blood pro-brain-type natriuretic peptide, anticardiolipin antibodies, proteinuria within the follow-up period, and blood lipids, especially in the week before delivery. This situation may prevent some of the predictive variables from being included in the model. In future studies, we may expand the cohort size, prolong the follow-up duration, and perform prospective validation, which might compensate for the limitations of the current study.

## Conclusion

This study leveraged LCCA, screened a subpopulation of PE patients at high risk of persistent PHTN, and identified some related risk factors, including older age, higher BMI, earlier PE onset, longer interval between births, higher incidence of abnormal pregnancy, and worse laboratory results. This study’s findings may help clinicians realize the severity of persistent PHTN, encourage patients to actively seek early medical advice, facilitate the early identification of high-risk PE women, and encourage precise monitoring and management of postpartum BP.

## Supplementary Information

**Additional file 1: Table S1.** Goodness-of-fit indicators for the five different class models. **Table S2.** Comparison of risk factors among the three clusters of PE patients. **Table S3.** Class assignment for a minority of PE patients.

## Data Availability

The original datasets of the present study are available from the corresponding author on reasonable request.

## Abbreviations

BMI: Body mass index
BP: Blood pressure
CVD: Cardiovascular disease
HDP: Hypertensive disorder of pregnancy
LCCA: Latent class cluster analysis
LPA: Latent profile analysis
MAP: Mean arterial pressure
OR: Odds ratios
PE: Preeclampsia
PHTN: Postpartum hypertension
