# Supplementary material for: Screening high-risk population of persistent postpartum hypertension in women with preeclampsia using latent class cluster analysis
Source: BMC Pregnancy Childbirth. 2022 Sep 6;22:687. doi: 10.1186/s12884-022-05003-4 (PMC9446580; doi:10.1186/s12884-022-05003-4)
Supplement: Supplementary file 1 — Additional file 1: Table S1. Goodness-of-fit indicators for the five different class models. Table S2. Comparison of risk factors among the three clusters of PE patients. Table S3. Class assignment for a minority of PE patients. [file 12884_2022_5003_MOESM1_ESM.docx]

**Supplementary Material**

**Screening high-risk population of persistent postpartum hypertension in women with preeclampsia using latent class cluster analysis**

Yuan-Yuan Li^1,2^, Jing Cao^1^, Jia-Lei Li^1^, Jun-Yan Zhu^1^, Yong-Mei Li^1^, De-Ping Wang^1^, Hong Liu^2^, Hai-Lan Yang^3^, Yin-Fang He^3^, Li-Yan Hu^4^, Rui Zhao^5^, Chu Zheng^6^, Yan-Bo Zhang^6,*^, Ji-Min Cao^1,*^

1. Key Laboratory of Cellular Physiology at Shanxi Medical University, Ministry of Education, and the Department of Physiology, Shanxi Medical University, Taiyuan, China

2. Department of Critical Care Medicine, The First Hospital of Shanxi Medical University, Taiyuan, China

3. Department of Maternity, The First Hospital of Shanxi Medical University, Taiyuan, China

4. Department of Obstetrics Gynecology, Shanxi Children's Hospital and Women Health Center, Taiyuan, China

5. Department of Clinical Laboratory, Shanxi Children's Hospital and Women Health Center, Taiyuan, China

6. Division of Health Statistics, School of Public Health, Shanxi Medical University, Taiyuan, China

^*^ Corresponding Authors. Email: [caojimin@sxmu.edu.cn](mailto:caojimin@sxmu.edu.cn) (J.M.C.); [sxmuzyb@126.com](mailto:sxmuzyb@126.com) (Y.B.Z.)

**Supplementary Tables**

**Table S1** Goodness-of-fit indicators for the five different class models

| Model | Log likelihood | df | BIC | ICL |
| --- | --- | --- | --- | --- |
| 2-cluster | −10381.8 | 82 | −21330.03 | −21479.49 |
| 3-cluster | −10164.82 | 120 | −21158.57 | −21332.86 |
| 4-cluster | −9724.35 | 74 | −19959.87 | −20140.6 |
| 5-cluster | −9673.522 | 84 | −19927.3 | −20111.99 |
| 6-cluster | −9611.505 | 94 | −19872.34 | −20086.91 |

**Table S2** Comparison of risk factors among the three clusters of PE patients

| Indicator variable | Risk factors | Cluster 1 (320) | Cluster 2 (368) | Cluster 3 (312) | *P* | Multiple comparisons |
| --- | --- | --- | --- | --- | --- | --- |
| 1. Maternal delivery age | Maternal delivery age | 30.23±4.29 | 30.17±4.92 | 31.10±5.29 | 0.024 | 3 > 2 |
| 2. Mean arterial pressure | Mean arterial pressure (MAP) (mmHg) | 112.30±7.44 | 119.58±11.31 | 129.60±12.96 | < 0.001 | 3 > 2 > 1 |
| 3. Drugs use | Beta blockers | 51 (15.94) | 18 (4.89) | 208 (66.67) | < 0.001 | 3 > 1 > 2 |
|  | Calcium antagonist | 8 (2.50) | 6 (1.63) | 104 (33.33) | < 0.001 | 3 > 2, 3 > 1 |
|  | dexamethasone | 0 (0.00) | 1 (0.27) | 52 (16.67) | < 0.001 | 3 > 2, 3 > 1 |
|  | MgSO4 | 2 (0.63) | 2 (0.54) | 103 (33.01) | < 0.001 | 3 > 2, 3 > 1 |
| 4. Medical history | Body mass index (BMI) before pregnancy | 131 (40.94) | 131 (35.60) | 130 (41.67) | 0.201 |  |
|  | Prior preeclampsia | 16 (5.00) | 23 (6.25) | 25 (8.01) | 0.299 |  |
|  | History of heart / kidney disease | 6 (1.88) | 2 (0.54) | 1 (0.32) | 0.078 |  |
|  | Family history of hypertension | 41 (12.81) | 47 (12.77) | 61 (19.55) | 0.021 | 3 > 2 |
|  | Number of abortions |  |  |  | 0.006 | 3 > 1 |
|  | 0 | 189 (59.06) | 212 (57.61) | 152 (48.72) |  |  |
|  | 1 | 95 (29.69) | 100 (27.17) | 102 (32.69) |  |  |
|  | 2 | 31 (9.69) | 38 (10.33) | 34 (10.90) |  |  |
|  | ≥ 3 | 5 (1.56) | 18 (4.89) | 24 (7.69) |  |  |
|  | Number of births |  |  |  | < 0.001 | 3 > 2, 3 > 1 |
|  | 0 | 220 (68.75) | 222 (60.33) | 149 (47.76) |  |  |
|  | 1 | 85 (26.56) | 124 (33.70) | 131 (41.99) |  |  |
|  | 2 | 14 (4.38) | 21 (5.71) | 25 (8.01) |  |  |
|  | ≥ 3 | 1 (0.31) | 1 (0.27) | 7 (2.24) |  |  |
|  | ≥ 10 years from the previous birth | 20 (6.25) | 45 (12.23) | 59 (18.91) | < 0.001 | 3 > 2 > 1 |
|  | PE was diagnosed before 32 weeks of gestation | 73 (22.81) | 110 (29.89) | 173 (55.45) | < 0.001 | 3 > 2, 3 > 1 |
| 5. Adverse pregnancy outcome | Preterm birth | 86 (26.88) | 227 (61.68) | 236 (75.64) | < 0.001 | 3 > 2 > 1 |
|  | Postpartum hemorrhage | 10 (3.13) | 28 (7.61) | 11 (3.53) | 0.010 | 2 > 1 |

**Table S2** (continued)

|  | Pericardial or pleural effusion | 0 (0.00) | 18 (4.89) | 20 (6.41) | < 0.001 | 3 > 1, 2 > 1 |
| --- | --- | --- | --- | --- | --- | --- |
|  | Placental abruption | 1 (0.31) | 62 (16.85) | 49 (15.71) | < 0.001 | 3 > 1, 2 > 1 |
|  | Low birth weight infants / fetal growth restriction | 12 (3.75) | 71 (19.29) | 71 (22.76) | < 0.001 | 3 > 1, 2 > 1 |
|  | HELLP syndrome | 4 (1.25) | 32 (8.70) | 34 (10.90) | < 0.001 | 3 > 1, 2 > 1 |
| 6. Blood cell and coagulation test | Platelet count | 5 (1.56) | 27 (7.34) | 33 (10.58) | < 0.001 | 3 > 1, 2 > 1 |
|  | Neutrophil count | 171 (53.44) | 194 (52.72) | 190 (60.90) | 0.068 |  |
|  | Monocyte count | 107 (33.44) | 120 (32.61) | 107 (34.29) | 0.898 |  |
|  | PT% | 93 (29.06) | 142 (38.59) | 133 (42.63) | 0.001 | 3 > 1, 2 > 1 |
|  | INR | 30 (9.38) | 66 (17.93) | 80 (25.64) | < 0.001 | 3 > 2 > 1 |
| 7. Liver and renal function | AST | 24 (7.50) | 57 (15.49) | 76 (24.36) | < 0.001 | 3 > 2 > 1 |
|  | ALT | 10 (3.13) | 28 (7.61) | 44 (14.10) | < 0.001 | 3 > 2 > 1 |
|  | ALB | 54 (16.88) | 171 (46.47) | 186 (59.62) | < 0.001 | 3 > 2 > 1 |
|  | Proteinuria |  |  |  | < 0.001 | 3 > 2 > 1 |
|  | 0 | 62 (19.38) | 42 (11.41) | 18 (5.77) |  |  |
|  | 1+ | 132 (41.25) | 88 (23.91) | 40 (12.82) |  |  |
|  | 2+ | 61 (19.06) | 62 (16.85) | 63 (20.19) |  |  |
|  | 3+ | 55 (17.19) | 145 (39.40) | 167 (53.53) |  |  |
|  | 4+ | 10 (3.13) | 31 (8.42) | 24 (7.69) |  |  |
|  | Serum creatinine | 5 (1.56) | 24 (6.52) | 31 (9.94) | < 0.001 | 3 > 1, 2 > 1 |
|  | Serum urea nitrogen | 2 (0.63) | 16 (4.35) | 36 (11.54) | < 0.001 | 3 > 2 > 1 |
| 8. Blood myocardial enzyme | Serum creatine kinase | 0 (0.00) | 48 (13.04) | 35 (11.22) | < 0.001 | 3 > 1, 2 > 1 |
| and electrolyte test | Serum lactate dehydrogenase | 0 (0.00) | 154 (41.85) | 156 (50.00) | < 0.001 | 3 > 1, 2 > 1 |
|  | Serum potassium | 0 (0.00) | 26 (7.07) | 16 (5.13) | < 0.001 | 3 > 1, 2 > 1 |
|  | Serum calcium | 0 (0.00) | 179 (48.64) | 178 (57.05) | < 0.001 | 3 > 1, 2 > 1 |

MAP, mean arterial pressure, expressed as median (25% quartile, 75% quartile), i.e., [M (P25, P75)]. BMI, body mass index. HELLP syndrome, hemolysis, elevated liver enzymes, low platelets syndrome. PT%, prothrombin activity (%). INR, international normalized ratio. AST, aspartate aminotransferase. ALT, alanine aminotransferase. ALB, albumin.

**Table S3** Class assignment for a minority of PE patients

| Y1 | Y2 | Y3 | Y4 | Y5 | Y6 | Y7 | Y8 | *P*(1\|*y*) | *P*(2\|*y*) | *P*(3\|*y*) | Cluster |
| --- | --- | --- | --- | --- | --- | --- | --- | --- | --- | --- | --- |
| 1.34 | −0.45 | −0.66 | 2.14 | −1.11 | 0.45 | −1.63 | −0.90 | 1 | 0 | 0 | 1 |
| −0.72 | −0.58 | −0.66 | −1.26 | 0.03 | −0.45 | 1.40 | 2.52 | 0 | 1 | 0 | 2 |
| 1.75 | −0.81 | −0.66 | 0.44 | −1.11 | 0.45 | −1.02 | −0.90 | 1 | 0 | 0 | 1 |
| −0.51 | －0.19 | −0.66 | −1.26 | 0.03 | 0.45 | −1.63 | 0.24 | 0 | 1 | 0 | 2 |
| 1.13 | −0.55 | 0.53 | 1.58 | 0.03 | −1.34 | −1.02 | −0.90 | 1 | 0 | 0 | 1 |
| −1.74 | −0.29 | 0.53 | −0.13 | −1.11 | −0.45 | 0.19 | −0.90 | 1 | 0 | 0 | 1 |
| −0.30 | 0.36 | 1.72 | −0.70 | 0.03 | −0.45 | 0.19 | −0.90 | 0 | 0 | 1 | 3 |
| −1.54 | −0.68 | −0.66 | −0.13 | −1.11 | 0.45 | −0.42 | −0.90 | 1 | 0 | 0 | 1 |
| 0.72 | 2.00 | −0.66 | 2.14 | 0.03 | −0.45 | −0.42 | −0.90 | 0 | 0 | 1 | 3 |

Y1–Y8 are standardized values of the 8 indicator variables. P(1|*y*), P(2|*y*), and P(3|*y*) indicate the probability of a class belonging (*y*). Cluster denotes for class assignment.
